# Microfibrillated Lignocellulose Enables the Suspension-Polymerisation of Unsaturated Polyester Resin for Novel Composite Applications

**DOI:** 10.3390/polym8070255

**Published:** 2016-07-11

**Authors:** Yutao Yan, Sabine Herzele, Arunjunai Raj Mahendran, Matthias Edler, Thomas Griesser, Bodo Saake, Jianzhang Li, Wolfgang Gindl-Altmutter

**Affiliations:** 1 Ministry of Education Key Laboratory of Wooden Material Science and Application, Beijing Key Laboratory of Wood Science and Engineering, MOE Engineering Research Centre of Forestry Biomass Materials and Bioenergy, Beijing Forestry University, Beijing 100083, China; yytao1988@163.com; 2 Kompetenzzentrum Holz GmbH, Altenbergerstrasse 69, A-4040 Linz, Austria; sabine.herzele@kplus-wood.at (S.H.); a.mahendran@kplus-wood.at (A.R.M.); 3 Department of Polymer Technology, University of Leoben, Otto Glöckel Strasse 2, A-8700 Leoben, Austria; matthias.edler@unileoben.ac.at (M.E.); thomas.griesser@unileoben.ac.at (T.G.); 4 Zentrum Holzwirtschaft, University of Hamburg, Leuschnerstrasse 91, D-21031 Hamburg-Bergedorf, Germany; bodo.saake@uni-hamburg.de; 5 Department of Materials Science and Process Engineering, BOKU–University of Natural Resources and Life Science Vienna, Konrad Lorenz Strasse 24, A-3430 Tulln, Austria

**Keywords:** microfibrillated cellulose, unsaturated polyester resin, suspension polymerization, composite reinforcement

## Abstract

A new route towards embedding fibrillated cellulose in a non-polar thermoset matrix without any use of organic solvent or chemical surface modification is presented. It is shown that microfibrillated lignocellulose made from cellulose with high residual lignin content is capable of stabilising an emulsion of unsaturated polyester resin in water due to its amphiphilic surface-chemical character. Upon polymerisation of the resin, thermoset microspheres embedded in a microfibrillated cellulose network are formed. The porous network structure persists after conventional drying in an oven, yielding a mechanically stable porous material. In an application experiment, the porous material was milled into a fine powder and added to the polyester matrix of a glass fibre-reinforced composite. This resulted in a significant improvement in fracture toughness of the composite, whereas a reduction of bending strength and stiffness was observed in parallel.

## 1. Introduction

The enormous potential of nanocellulose, i.e., nano-scale cellulose fibrils or crystals, towards novel composite materials with improved or wholly new functionalities is widely acknowledged [1,2,3,4,5,6]. Among the challenges to be overcome on the way to realising this potential for industrial applications, the lack of inherent surface-chemical compatibility between essentially hydrophilic cellulose and many hydrophobic polymers and organic solvents is of high relevance. Chemical surface modification is capable of fine-tuning the degree of hydrophobicity of nanocellulose, and highly capable of producing excellent dispersions of nanocellulose in hydrophobic media [7]. However, even though highly efficient when dealing with small quantities of material in the laboratory, the up-scaling of chemical modification raises questions concerning its feasibility at reasonable cost. Drying, another option for transferring nanocellulose from its native aqueous state to non-aqueous environments, is energy-intensive and of limited efficiency with regard to preserving the nano-scale morphology of nanocellulose [8]. Thus, the challenges of interfacial compatibility and the lack of a technically- and economically efficient drying method need to be overcome with regard to a widespread utilisation of nanocellulose in thermoplastic or thermoset matrix composites. Compared to thermoplastic matrices, literature on nanocellulose-thermoset composites is relatively sparse. Procedures described in the latter case usually involve the infiltration of an existing nanofiber network with reactive oligomers, which is followed by solvent evaporation and curing [9,10,11,12].

Emulsions were proposed as novel and elegant routes to the compounding of nanocellulose with hydrophobic media [13]. For example, a stable nanocomposite dispersion was obtained by miniemuelsion polymerisation of acrylic monomers in the presence of cellulose nanocrystals and a silane coupling agent [14]. Polystyrene-nanocellulose composite microbeads were polymerised from emulsion [15]. Polymethylmethacrylate-nanocellulose composites were successfully obtained in suspension polymerization [16,17]. Bacterial cellulose hydrophobised by means of silylation or acetylation, respectively, was used to stabilise medium and high internal phase water-in-acrylated soybean oil emulsions in the production of fully bio-based macroporous thermosetting cellulose nanocomposite [18]. Microbeads and hollow microcapsules were obtained by self-assembly of pickering magneto-responsive nanocellulose [19]. Finally, a route for the compatibilisation of aqueous dispersions of cellulose nanofibrils with a non-polar polystyrene matrix was introduced using an emulsion route involving non-ionic surfactants [20]. A comparable approach was recently realised for chitin nanofibers and an acrylic resin [21]. In this study, an emulsion of resin was produced and stabilised by chitin nanofibers. Instead of polymerisation directly in suspension, however, filtration was performed, yielding a resin-nanofibre compound which was subsequently dried and cured to obtain a solid nanofiber-reinforced polymer sheet.

Recently, it was shown that the surface-chemical amphiphilicity of lignin benefits the dispersion of nanocellulose in non-polar anorganic media [22,23,24]. Using the example of styrene monomer, lignocellulose nanofiber-reinforced composites with superior impact strength were produced from polystyrene-lignocellulose composite microspheres obtained in suspension polymerization [25]. Notably, neither chemical surface modification, nor drying or transfer to organic solvent were used in this study in order to arrive at a high-performance composite of essentially polar nanocellulose and polystyrene.

In the present study, we show that—in a similar way—microfibrillated lignocellulose stabilises emulsions of unsaturated polyester resin in water which, upon polymerisation, produces polyester-lignocellulose composite microspheres. A potential application perspective of this material as additive to the matrix of fibre-reinforced polyester composites is presented.

## 2. Materials and Methods

### 2.1. Production of Microfibrillated Lignocellulose

The production of microfibrillated lignocellulose used in the present study is described in detail in Ref. [25]. Briefly, beech wood chips were partly delignified in an autoclave by means of a water/ethanol (50/50 weight) mixture containing 0.75 wt % H_2_SO_4_ as a catalyst. The temperature during the 90 min treatment was kept at 170 °C, resulting in a pressure of 1.5 MPa. After depressurisation and cooling the treated material was repeatedly washed and disintegrated into nano-scale fibrillary materials by subsequent treatment with a disc-refiner, a Masuko supermasscollider, and an APV high-pressure homogeniser. The material produced this way is termed microfibrillated lignocellulose (MFLC). For reference purposes, standard microfibrillated cellulose (MFC) was obtained from the University of Maine and was homogenized before use (15 cycles, 80 MPa).

### 2.2. Suspension Polymerisation

The unsaturated polyester resin used was Palatal U 569 TV-01 obtained from R&G Composite Technology, Waldenbuch, Germany. Methylethylketoneperoxide hardener (2% *w*/*w*) was added before use. The resin was dispersed in 90 mL of aqueous fibril suspension containing 0.5% (*w*/*w*) fibrils by means of an ultra-turrax device. The amounts of resin added were 1, 2, 5, and 10 mL. For polymerisation, 6 mL of the mixture was enclosed in a 10 cm^−3^ sealable polytetrafluoroethylene reaction vessel and left in an oven at 103 °C for 2 h. After de-pressurisation and cooling the reaction product was dried conventionally in the same oven at 103 °C. For characterisation and further experiments, the dry reaction product was either milled to rectangular shape using a razor blade or disintegrated into fine powder by means of a ceramic mortar.

### 2.3. Fibre-Reinforced Composites

The same unsaturated polyester resin with 2% hardener mentioned above was either used as received, or modified by adding 1% (*w*/*w*) of cellulose-polyester powder. Plain weave glass fibre mats with an area weight of 163 g·m^−2^ obtained from R&G Composite Technology, Germany, were impregnated with a roller and stacked to 15 plies. Curing was performed in a press operated at room temperature and a platen distance of 2.5 mm, resulting in a volumetric fibre content of roughly 25% in the composite sheets. Before characterisation the composites were post-cured in an oven at 80 °C for 12 h as recommended by the manufacturer. In parallel, samples of resin were also cured without adding glass fibre in order to characterise pure resin properties.

### 2.4. Characterisation

The surface chemistry of dry microfibrillated cellulose was characterised by means of X-ray photoelectron spectroscopy (XPS) using a K-Alpha spectrometer (Thermo Fischer Scientific, Waltham, MA USA). Survey scans were done with a pass energy of 200 eV and a step size of 1.0 eV. High resolution scans of the carbon peak region were done with a pass energy of 50 eV and a step size of 0.1 eV. All spectra were normalized to the Au peak. The average chemical composition was calculated from wide scan spectra. The peaks in the high resolution spectra were fitted using a Gaussian/Lorentzian mixed function employing Shirley background correction.

MFLC was characterised with atomic force microscopy (AFM). AFM was done in tapping mode using a Dimension Icon AFM (Bruker, Karlsruhe, Germany) and standard tapping mode probes by the same manufacturer. Sample preparation for AFM was accomplished by placing a drop of the respective 0.001 wt % aqueous fibril suspension onto freshly cleaved mica, and evaporating the water at ambient conditions.

Liquid emulsions of unsaturated polyester resin were observed with a Zeiss Axioplan fluorescence microscope using Nile Red, Nile Blue and Calcoflour white stain. SEM of dry polymerised material was carried out using a Quanta™ 250 scanning electron microscope (FEI Europe B.V., Vienna, Austria) with a Shottky field emission gun after sputter coating the samples with gold.

Mechanical characterisation was carried out in three-point bending tests with a Zwick-Roell 20 kN universal testing machine equipped with a 20 kN load cell. Testing was done at a free tested sample length of 60 mm and a cross-head speed of 10 mm·min^−1^. Impact bending was performed on a Zwick-Roell instrumented Charpy 5N impact pendulum at a support distance of 60 mm (i.e., tested length). For both tests, 10 replicate samples with a total length of 100 mm and a width of 10 mm were used. Interlaminar shear strength testing was carried out in short beam shear testing according to ASTM (American Society of the International Association for Testing and Materials) D2344.

Thermogravimetric analysis (TGA) of unmodified and modified cured polyester resin was done with an STA 409PC/PG (Netzsch, Selb, Germany). Using aluminium crucibles 10 mg of sample were analysed in nitrogen atmosphere at a heating rate of 5 °C·min^−1^.

## 3. Results and Discussion

Important characteristics of microfibrillated lignocellulose (MFLC) and microfibrillated cellulose (MFC) are summarised in Table 1. Compared to standard MFC derived from bleached pulp, MFLC contains substantial amounts of lignin and hemicellulose. Even though polysaccharide just as cellulose, hemicellulose differs significantly in structure and composition.

**Figure 1:**
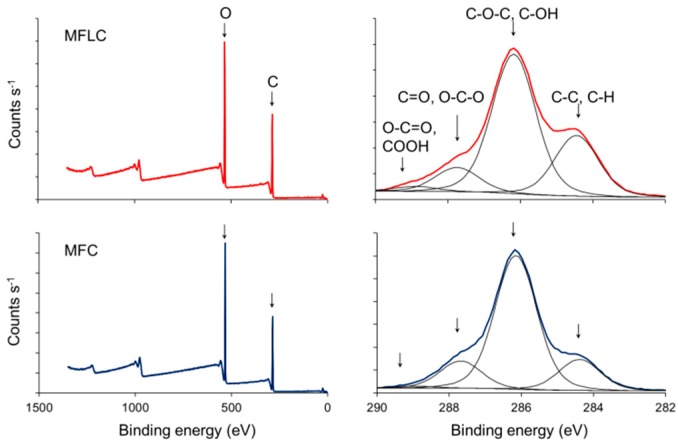
Detailed results of survey scans and high-resolution scans of the carbon peak from X-ray photoelectron spectroscopy (XPS) of MFLC and MFC.

Hemicellulose is a branched molecule containing glucose just as cellulose, but also other monomer units such as xylan. It also disposes of accessible functional moieties other than hydroxyl groups, e.g., acetyl functions. This difference in structure and composition also entails differences in surface chemistry, which manifest themselves in terms of improved dispersion of high-hemicellulose MFC, termed fibrillated holocellulose, in organic solvent [26]. Lignin, on the other hand, is an amorphous aromatic polymer containing distinctly hydrophilic moieties such as hydroxyl groups, but also clearly hydrophobic functionalities, which, e.g., translate to reduced wettability with water in MFLC compared to MFC [27]. Such a change in surface-chemical properties is confirmed by XPS measurements (Figure 1).

Survey scans of dry material indicate a carbon/oxygen ratio of 1.57 for MFC as opposed to 1.81 for MFLC, which agrees well with the substantial lignin content in the MFLC sample compared to MFC, which is almost free of lignin. A detailed scan of the carbon peak shown in Figure 1 provides further indications of reduced hydrophilicity in MFLC. Very clearly, there is a more abundant presence of C–C and C–H moieties in MFLC compared to MFC in relation to the main peak representing C–O–C and C–OH moieties, respectively.

**Figure 2:**
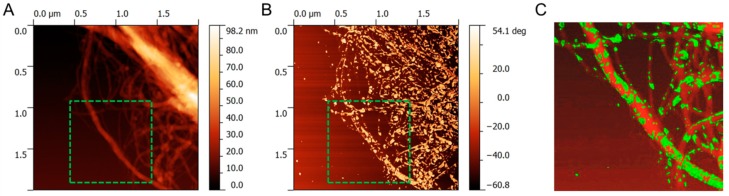
Atomic force microscopy (AFM) images of MFLC. (**A**) Height image, (**B**) phase image, (**C**) superimposed and digitally enhanced image of regions marked with a green dash square in the height and phase images. In image (**C**), red signal corresponds to height and green signal corresponds to phase.

AFM images shown in Figure 2 confirm heterogeneity of MFLC. The appearance of MFLC in the height image (Figure 2A) shows individual fibrils and fibril agglomerations with occasionally occurring granular structures present on their surface. The corresponding phase image (Figure 2B) reveals heterogeneous material properties of the fibrillary material. Superposition and digital enhancement of the height and the phase image clearly reveals that patches of material other than the bulk of the fibrils are present along the fibril structure (Figure 2C). Due to the distinct contrast seen in the phase image it is proposed that the substance occasionally covering the fibrils is residual lignin and hemicellulose.

**Figure 3:**
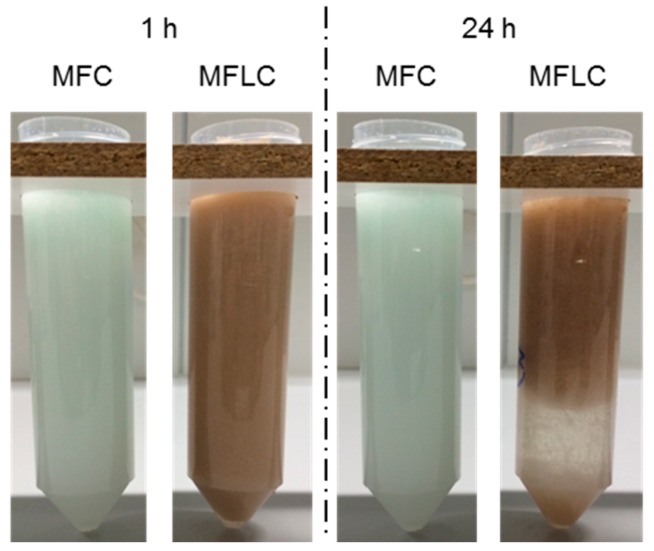
Optical appearance of emulsions of liquid unsaturated polyester resin in water stabilised by 0.5% MFC and MFLC, observed 1 and 24 h after mixing, respectively (tube diameter is 20 mm).

Based on the differences in fibril bulk- and surface-chemistry, presumably due to residues of lignin and hemicellulose covering the surface of MFLC fibrils, it was expected that MFLC would be better suited to stabilise emulsions of hydrophobic liquids in water compared to MFC (Figure 3).

**Figure 4:**
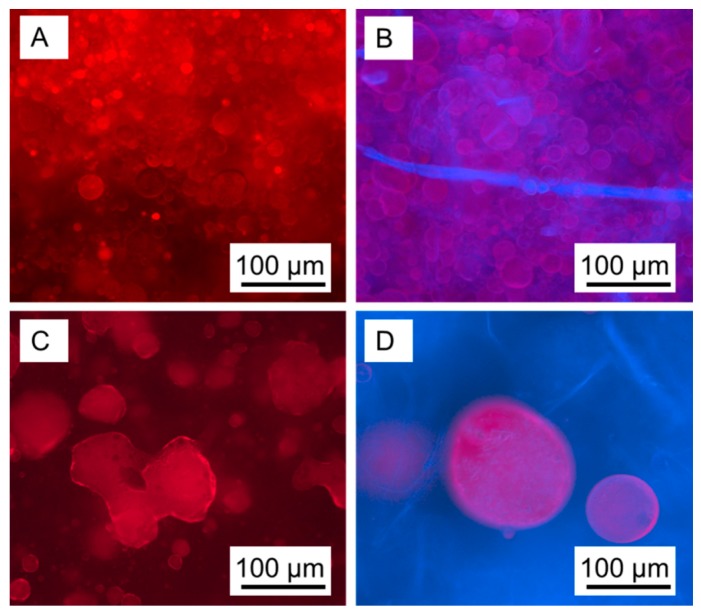
Visible-light (**A**,**C**) and fluorescence (**B**,**D**) microscopy of unsaturated polyester resin in water emulsions stabilised by MFLC (**A**,**B**) and MFC (**C**,**D**). Red colour corresponds to unsaturated polyester and blue colour corresponds to celluloses.

Initially, both systems are stable macroscopically, but after 24 h significant differences are present. Firstly, unsaturated polyester resin appears to agglomerate on the surface of the MFC variant, which is not seen in the MFLC variant. Secondly, the volume taken-up by brown MFLC in the container is reduced considerably as water containing occasional agglomerates of MFLC occupies a discrete region at the bottom of the container. No indications for the presence of unsaturated polyester resin were found in this region, which is why it is assumed that the resin is still present in emulsified state. This assumption is confirmed by microscopic investigations of emulsions shown in Figure 4. In visible light mode, unsaturated polyester resin droplets appear in bright red against a darker background. The droplet size is between 10 and 30 μm in the MFLC variant (Figure 4A), whereas considerably larger droplets and agglomerations of irregular shape with diameters between 40 and 100 μm are found in the MFC-stabilised variant (Figure 4C). With regard to differences in droplet size, images taken in fluorescence mode show the same information as in Vis mode. The colour contrast, however, is now different, with resin in reddish colour against a blueish background due to fibrous material. Apart from a few larger elements, no individual fibrous elements can be discerned. The MFLC stabilised variant appears to be rather homogeneous (Figure 4B), with a high density of small droplets.

**Figure 5:**
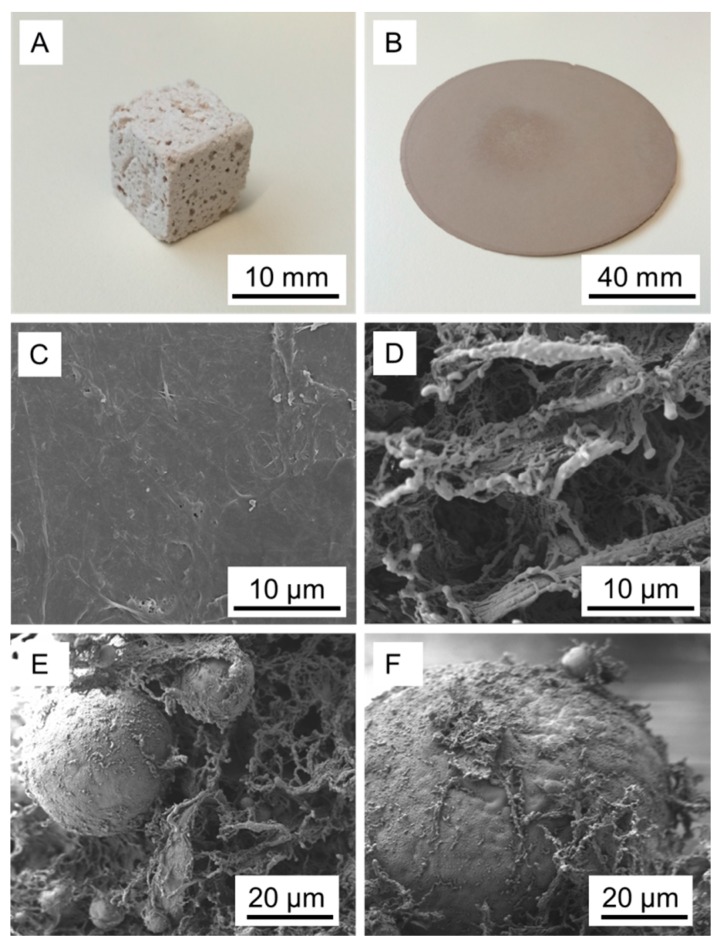
Optical appearance of an oven-dried porous polyester-MFLC compound (**A**) compared to an MFLC nanopaper (**B**); SEM images of an MFLC nanopaper surface (**C**) compared to the network-like structure of MFLC dried in the presence of unsaturated polyester (**D**). SEM images of cured spheres of polymer embedded in MFLC network (**E**,**F**).

By contrast, much fewer but significantly larger droplets enclosed in the fibril suspension are seen in the MFC stabilised variant (Figure 4D). In summary, both results shown in Figure 3 and Figure 4 confirm the initial assumption that MFLC may be suitable for stabilising unsaturated polyester resin-in-water emulsions, which is not the case to a comparable degree for MFC. The different capability of MFC and MFLC in terms of stabilising emulsions directly affects the outcome of attempts at polymerising the unsaturated polyester resin from emulsion (Figure 5). None of the MFC stabilised variants could be polymerised as the liquid taken from polymerisation containers remained sticky. Only after drying and evaporating all water in an oven the polyester finally became solid, incorporating MFC. In strong contrast, attempts at curing unsaturated polyester resin from emulsions stabilised with MFLC were successful. After polymerisation a sponge-like water-saturated material was obtained, which transformed into a solid mass upon drying for the variants with low and medium resin content. By contrast, the variant with highest resin content (10 mL resin in 90 mL aqueous MFLC suspension) could be easily dried conventionally without collapse (Figure 5A), resulting in a density of 250 g·cm^−3^. The fact that such a high degree of porosity is maintained after drying is remarkable in view of the normal drying behaviour of fibrillated cellulose. The same MFLC dried from aqueous suspension in absence of unsaturated polyester forms a sheet of nanopaper (Figure 5B) with density >1 g·cm^−3^, as is well known for nanocellulose conventionally dried from water [28]. Thus, in the SEM MFLC dried from water appears to be solid, showing extremely sparse porosity (Figure 5C). Contrarily, dried in the presence of cured unsaturated polyester, MFLC retains a high amount of fibrillary structure and porosity (Figure 5D). Typically, the diameter of individual fibrils is in the range between 50 and 200 nm, with occasional agglomerations up to 500 nm in diameter. Spheres of cured polymer with diameters between 10 and 80 μm are embedded in this network of MFLC fibrils (Figure 5E). While a large portion of MFLC is present in an independent network between polymer spheres, a small fraction of MFLC appears to be attached to the surface of individual polymer spheres, which it partially covers in a network structure (Figure 5F). The fact that MFLC not only is present in spaces between polymer spheres, but also appears to be attached to the surface of the spheres indicates that MFLC indeed shows a certain degree of surface-chemical affinity to the polymer, similarly as observed for chemically hydrophobised nanocellulose and polystyrene [15], and MFLC and polystyrene [25], respectively.

**Figure 6:**
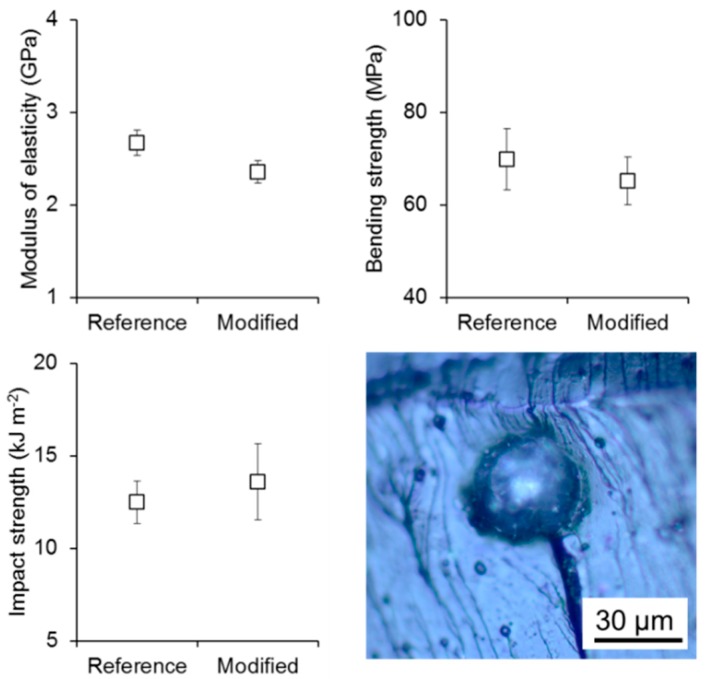
Results of the mechanical characterisation of sheets obtained from cured unsaturated polyester resin. While the unsaturated polyester resin was used as received in the reference material, the modified variant contains 1% filler consisting of polyester microspheres and MFLC fibrils at a ratio of 95/5. The microscope image shows the fracture surface of three-point bending samples, with the inset showing one polyester microsphere located at the fracture surface.

**Figure 7:**
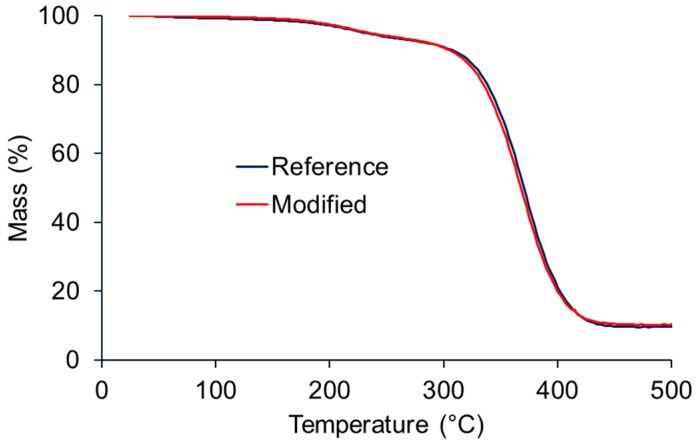
Results of the characterisation of sheets obtained from cured unsaturated polyester resin by means of thermogravimetric analysis (TGA).

**Figure 8:**
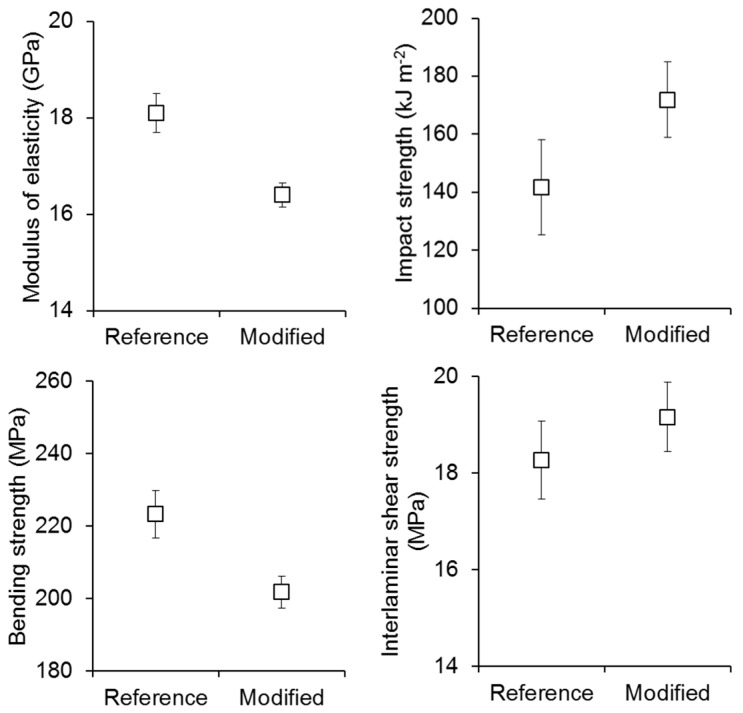
Results of the mechanical characterisation of glass-fibre reinforced polyester composites (unsaturated polyester resin was used as received in the reference material, the modified variant contains 1% filler consisting of polyester microspheres and MFLC fibrils).

Thus a new method is established to achieve a compound of a thermoset resin and fibrillated cellulose, notably without using chemical surface modification, transfer to organic solvent, or sophisticated drying methods. Using the approach presented in the present study, a porous sub-micron MFLC network structure containing 95% polyester spheres is obtained. One potential application of this novel type of material may be seen in disintegrating the porous network into fine powder, and using it as an additive for thermoset resins, profiting from a potential reinforcement effect of sub-micron MFLC. The results of such an experiment are shown in Figure 6, Figure 7 and Figure 8.

Overall, the addition of 1% polyester spheres/MFLC fibrils resulted in an approximate net content of 0.05% MFLC fibrils in the resin. The effect of polyester spheres/MFLC on the mechanics of the polymer shows a decrease in modulus of elasticity and bending strength, whereas a slight improvement in impact strength is seen, which is not significant in a statistical sense. These changes, even though relatively small, agree well with general principles observed for a large variety of particulate fillers [29]. Furthermore, in a study using unmodified and surface-hydrophobised cellulose nanofiller for unsaturated polyester resin, similar effects, i.e. different trends for strength and impact characteristics, respectively, were observed [30]. In Ref. [30], the unmodified variant proved more efficient with regard to improving fracture toughness, whereas the surface hydrophobised variant was more beneficial to stiffness and strength. The light-micrograph shown in Figure 6 indicates that MFLC-coated polyester spheres detach from the surrounding matrix during fracture, where the MFCL-coating serves as a point of weakness and pre-determined crack path. Thus the presence of MFLC-coated polyester spheres leads to an enlarged fracture surface, which explains the slight improvement in toughness observed. However, in-turn, the weak interface reduces the strength of the polyester sphere-filled polymer films (Figure 6).

The thermal stability of the polyester sheets both with and without addition of polyester microspheres as revealed by TGA is shown in Figure 7. No significant difference was observed between the two variants tested, confirming that the addition of MFLC-coated microspheres did not affect thermal stability of the cured resin.

When using MFLC-coated polyester spheres for the modification of an unsaturated polyester matrix in a glass-fibre-reinforced composite, clear effects on mechanical performance are observed (Figure 8). Similar to pure polymer sheets, the mechanical performance of the composite in three-point bending is negatively affected by the addition of polyester/MFLC spheres. The modulus of elasticity is reduced by 10% and a comparable loss in bending strength is observed. Again it is proposed that this negative effect of filler addition is caused by the weak interface between microspheres added, and the bulk polyester matrix. As expected from the positive impact testing results with polyester sphere-filled polymer films, unnotched Charpy impact pendulum testing reveals a clear improvement of fracture toughness by 20% also in the case of a glass fibre-reinforced composite (Figure 8). Also interlaminar shear strength is slightly improved by 5%. Thus the addition of particulate filler to the matrix of glass fibre-reinforced polyester composites resulted in improved interlaminar properties expressed in terms of shear strength and impact performance. The same observation is made when nano-particulate filler is added to the thermoset matrix of carbon fibre-reinforced composites [31,32,33,34,35]. Therefore, the trends of changes in properties with addition of particulate filler shown in Figure 6 and Figure 8 agree well with literature.

## 4. Conclusions

The results presented above demonstrate that MFLC is capable of stabilising emulsions of unsaturated polyester resin in water. This enables the direct polymerisation of the resin in suspension, leading to a porous fibrillary compound of polyester microspheres and micro-fibrillar MFLC. One potential application route of this new material in terms of modifying the matrix polymer in glass fibre reinforced composites was examined and a positive effect on impact performance was obtained, whereas bending strength and stiffness were diminished.

## Figures and Tables

**Table 1:** Chemical composition of microfibrillated lignocellulose (MFLC) compared to microfibrillated cellulose (MFC) [25].

Type of Material	Glucose	Xylan	Lignin	Crystallinity
MFC	99.9	0.0	0.1	71
MFLC	62.0	14.1	14.0	70
